# Supplementary material for: Biogeographical and phylogenetic constraints on horizontal gene transfer and genome evolution in Streptomyces
Source: Microbiol Spectr. 2025 Dec 17;14(2):e02958-25. doi: 10.1128/spectrum.02958-25 (PMC12889140; doi:10.1128/spectrum.02958-25)
Supplement: Supplemental material — Tables S1 to S3. [file spectrum.02958-25-s0001.docx]

Supplementary Table 1. Assembly statistics of all draft genomes

| **Name** | **Cluster** | **Size (Mbp)** | **N50** | **Number of contigs** |
| --- | --- | --- | --- | --- |
| RH45 | RH-PR | 7.49 | 28462 | 475 |
| RH79 | RH-PR | 7.49 | 40914 | 365 |
| RH155 | RH-PR | 7.82 | 29432 | 538 |
| RH185 | RH-PR | 7.51 | 122364 | 173 |
| RH254 | RH-PR | 7.81 | 34852 | 455 |
| WA1 | WA-PR | 8.35 | 194417 | 92 |
| WA13 | WA-PR | 8.19 | 122969 | 148 |
| WA22 | WA-PR | 8.17 | 172815 | 112 |
| WA53 | WA-PR | 8.04 | 162578 | 137 |
| RH34 | RH-GR | 8.23 | 26146 | 630 |
| RH195 | RH-GR | 8.64 | 25829 | 621 |
| RH206 | RH-GR | 8.63 | 24347 | 671 |
| RH207 | RH-GR | 8.64 | 21115 | 786 |
| WA1002 | WA-GR | 8.64 | 20023 | 893 |
| WA1063 | WA-GR | 8.85 | 15367 | 1180 |
| WA1064 | WA-GR | 8.7 | 136483 | 175 |
| WA1071 | WA-GR | 8.75 | 11721 | 1401 |

Supplementary Table 2. Average Nucleotide Identity (ANI) between draft genomes analyzed in this dataset. ANI values between pairs of *griseus* strains are bolded and ANI values between *pratensis* strains are italicized.

|  | RH45 | RH79 | RH155 | RH185 | RH254 | WA1 | WA13 | WA22 | WA53 | RH34 | RH195 | RH206 | RH207 | WA1002 | WA1063 | WA1064 | WA1071 |
| --- | --- | --- | --- | --- | --- | --- | --- | --- | --- | --- | --- | --- | --- | --- | --- | --- | --- |
| RH45 |  |  |  |  |  |  |  |  |  |  |  |  |  |  |  |  |  |
| RH79 | **99.96** |  |  |  |  |  |  |  |  |  |  |  |  |  |  |  |  |
| RH155 | **99.27** | **99.26** |  |  |  |  |  |  |  |  |  |  |  |  |  |  |  |
| RH185 | **99.92** | **99.95** | **99.26** |  |  |  |  |  |  |  |  |  |  |  |  |  |  |
| RH254 | **99.26** | **99.24** | **99.94** | **99.18** |  |  |  |  |  |  |  |  |  |  |  |  |  |
| WA1 | **93.85** | **93.87** | **93.88** | **93.88** | **93.84** |  |  |  |  |  |  |  |  |  |  |  |  |
| WA13 | **93.86** | **93.96** | **93.92** | **93.93** | **93.87** | **99.64** |  |  |  |  |  |  |  |  |  |  |  |
| WA22 | **93.83** | **93.89** | **93.86** | **93.88** | **93.84** | **99.72** | **99.62** |  |  |  |  |  |  |  |  |  |  |
| WA53 | **93.94** | **94** | **93.91** | **93.99** | **93.86** | **99.61** | **99.58** | **99.68** |  |  |  |  |  |  |  |  |  |
| RH34 | 85.06 | 84.76 | 84.85 | 84.96 | 85.07 | 84.63 | 84.52 | 84.52 | 84.54 |  |  |  |  |  |  |  |  |
| RH195 | 84.84 | 84.79 | 84.86 | 84.87 | 84.72 | 84.83 | 84.74 | 84.68 | 84.77 | *94.3* |  |  |  |  |  |  |  |
| RH206 | 84.8 | 84.78 | 84.8 | 84.82 | 84.78 | 84.84 | 84.76 | 84.73 | 84.84 | *94.27* | *99.94* |  |  |  |  |  |  |
| RH207 | 84.84 | 84.83 | 84.84 | 84.84 | 84.76 | 84.81 | 84.7 | 84.7 | 84.85 | *94.26* | *99.92* | *99.9* |  |  |  |  |  |
| WA1002 | 84.91 | 84.82 | 84.76 | 84.81 | 84.85 | 84.6 | 84.62 | 84.64 | 84.71 | *94.07* | *96.66* | *96.7* | *96.66* |  |  |  |  |
| WA1063 | 84.93 | 84.87 | 84.86 | 84.88 | 84.91 | 84.68 | 84.69 | 84.73 | 84.83 | *93.9* | *96.48* | *96.53* | *96.49* | *99.11* |  |  |  |
| WA1064 | 84.89 | 84.82 | 84.74 | 84.83 | 84.81 | 84.72 | 84.69 | 84.62 | 84.68 | *94.09* | *96.78* | *96.75* | *96.79* | *99.19* | *99.24* |  |  |
| WA1071 | 84.94 | 84.88 | 84.78 | 84.9 | 84.82 | 84.79 | 84.72 | 84.74 | 84.77 | *93.96* | *96.47* | *96.52* | *96.48* | *99.8* | *99* | *98.89* |  |

Supplementary Table 3. Gene counts for all categories shown in Fig. 5. The gain/loss (G/L) ratio for each node (N) and tip corresponds with the annotations in the figure. The table shows the raw data used to calculate the ratio and a separate column documenting gene duplications.

| ***Node or Tip*** | ***Loss*** | ***Gain*** | ***G/L ratio*** | ***Duplications (counted in Gain)*** |
| --- | --- | --- | --- | --- |
| RH195 | 986 | 898 | 0.91 | 5 |
| N10 | 112 | 858 | 7.66 | 18 |
| RH206 | 979 | 881 | 0.90 | 1 |
| N6 | 1135 | 386 | 0.34 | 34 |
| RH207 | 1765 | 2519 | 1.43 | 7 |
| N3 | 194 | 259 | 1.34 | 122 |
| WA1002 | 2341 | 1551 | 0.66 | 14 |
| N14 | 667 | 585 | 0.88 | 17 |
| WA1063 | 1562 | 860 | 0.55 | 49 |
| N11 | 133 | 709 | 5.33 | 22 |
| WA1064 | 2342 | 1547 | 0.66 | 37 |
| N7 | 703 | 613 | 0.87 | 256 |
| WA1071 | 3037 | 2950 | 0.97 | 26 |
| RH34 | 769 | 949 | 1.23 | 254 |
| RH185 | 3670 | 2010 | 0.55 | 1 |
| N15 | 300 | 352 | 1.17 | 1 |
| RH155 | 2000 | 497 | 0.25 | 3 |
| N12 | 260 | 706 | 2.72 | 17 |
| RH254 | 2321 | 935 | 0.40 | 5 |
| N8 | 12 | 853 | 71.08 | 200 |
| RH45 | 3006 | 2036 | 0.68 | 2 |
| N4 | 369 | 595 | 1.61 | 473 |
| RH79 | 2748 | 2730 | 0.99 | 4 |
| WA13 | 1720 | 918 | 0.53 | 12 |
| N13 | 271 | 495 | 1.83 | 20 |
| WA1 | 1738 | 1044 | 0.60 | 48 |
| N9 | 68 | 588 | 8.65 | 19 |
| WA22 | 2319 | 1875 | 0.81 | 21 |
| N5 | 254 | 560 | 2.20 | 414 |
| WA53 | 2456 | 2580 | 1.05 | 38 |
